# Severe low cerebral oximetry in difficult cardiopulmonary bypass weaning of low body-weight infant: a case report and literature review

**DOI:** 10.1186/s12871-020-01071-1

**Published:** 2020-06-27

**Authors:** Xuechao Hao, Wei Wei

**Affiliations:** Department of Anesthesiology, The Research Unit of Perioperative Stress Assessment and Clinical Decision (2018RU012), Chinese Academy of Medical Sciences, West China Hospital, Sichuan University, 610041, Chengdu, People’s Republic of China

**Keywords:** Cerebral oximetry, Cardiopulmonary bypass, Low body-weight infant, Vasoconstrictor

## Abstract

**Background:**

For infants undergoing complex cardiac surgery, hemodynamic management after cardiopulmonary bypass (CPB) is challenging because of severe myocardial edema, vasomotor dysfunction and weak tolerance to a change in blood volume. More importantly, the lack of availability of equipment for advanced monitoring, such as transesophageal echocardiography or transthoracic echocardiography, restricts the accurate assessment of hemodynamics.

**Case presentation:**

This is a case of severe hypotension and non-detectable pulse oxygen saturation (SpO_2_) after CPB in a low-weight infant who had normal blood pressure and oxygen saturation before surgery. Epinephrine and milrinone were administered with cerebral oximetry monitoring rather than blood pressure measurements because cerebral oximetry was more responsive to treatment than blood pressure. Under the guidance of cerebral oximetry, the infant was successfully weaned from CPB and recovered after surgery without adverse neurological events.

**Conclusions:**

For infants who develop refractory hypotension and failure in SpO_2_ monitoring during the CPB weaning period, cerebral oximetry provides an index for assessing brain perfusion and valuable guidance for appropriate inotropic treatment.

## Background

For an infant undergoing complex cardiac surgery, hemodynamic management after cardiopulmonary bypass (CPB) is challenging because of severe myocardial edema, vasomotor dysfunction and weak tolerance to a change in blood volume [1]. More importantly, lack of availability of equipment for advanced monitoring, such as transesophageal echocardiography or transthoracic echocardiography, restricts the accurate assessment of hemodynamics [2]. This case describes the emergency application of cerebral oximetry in directing hemodynamic management in a low-weight infant who developed severe hypotension and a low saturation of blood oxygen after CPB. This report describes an algorithm of hemodynamic management using cerebral oximetry as the main monitoring tool during cardiac surgery with CPB in infants, especially during the CPB weaning period.

## Case presentation

A female infant aged 2 months, weight 2.7 kg, height 50 cm, diagnosed with ventricular septal defect (VSD), atrial septal defect (ASD) and severe pulmonary hypertension was scheduled for VSD repair under CPB. The preoperative TTE examination revealed a VSD of 9 mm and an ASD of 8 mm. The past medical or family history was unremarkable.

General anesthesia was induced by 0.5 mg midazolam, 5 μg fentanyl, 5 mg rocuronium and 7% sevoflurane. An endotracheal tube with an inner diameter of 3 mm was intubated using direct laryngoscope. After tracheal intubation, the infant was ventilated to normocapnia with an inspired oxygen fraction 0.5 by a fresh gas flow of 2 L/min of oxygen and air. Anesthesia was maintained with sevoflurane inhalation, intravenous infusion of remifentanil and injection of fentanyl and rocuronium as required. Invasive arterial blood pressure was continuously monitored through the left radial artery. The patient had a 5F double-lumen catheter placed in the right internal jugular vein for central venous pressure monitoring and medication. The arterial blood gas analysis after intubation (before surgery) was: pH 7.34, carbon dioxide partial pressure (PCO_2_) 39.5 mmHg, oxygen partial pressure (PO_2_) 218.8 mmHg and haemoglobin 82.4 g/L. TEE monitoring was not performed due to no available probe for an individual weighing less than 3 kg. Arterial blood pressure was 64/40 mmHg, and the SpO_2_ signal was good and the reading was 100% prior to surgery. Anesthesia and surgical repair were performed routinely. The operative site was exposed via a median sternotomy, then CPB was built successfully after injection of 1125 U heparin and cannulation at the aorta root, superior vena cava and inferior vena cava, with infusion of cardioplegia solution containing potassium. The core body temperature was maintained at 32 ~ 33 °C during CPB. The ASD and VSD were patched during 88 min of aortic cross-clamp period. The results of arterial or venous blood gas analysis during surgery are shown in *Supplementary file Table* *2**.*

After the aortic clamp was released, sinus rhythm was restored spontaneously at a rate of 120 to 130 beats per minute and the electrocardiogram showed ST segment elevation. Intravenous infusions of epinephrine and nitroglycerin were administered at a dose of 0.05 μg/(kg·min) and 0.5 μg/(kg·min), respectively. When bypass withdrawal was attempted, sustained hypotension and low saturation of blood oxygen occurred. The systolic blood pressure ranged between 40 ~ 50 mmHg and a SpO_2_ value at the finger could not be detected. An oximetry probe was attached to the ear of the infant for SpO2 monitoring, but no signal was detectable. The arterial blood gas analysis revealed a PaO_2_ of 35.4 mmHg, PaCO_2_ of 43.2 mmHg Hb and a hemoglobin concentration of 100.5 g/L. There was severe myocardial edema and this made the size of the heart exceed the pericardial cavity. The rate of the epinephrine intravenous infusion was increased to 0.1 μg/(kg·min), but no significant increase in blood pressure occurred. A systolic blood pressure below 50 mmHg is insufficient for satisfactory perfusion of body organs. As CPB had been completed and the aortic cannula removed, pressure detection at aortic root was not performed. Cerebral oximetry (Engin Bio-medical Electronics Co., Ltd., Suzhou, China) was applied to the left side to evaluate cerebral perfusion and revealed a cerebral tissue oxygen saturation (SctO_2_) of 35.7%.

**Fig. 1:**
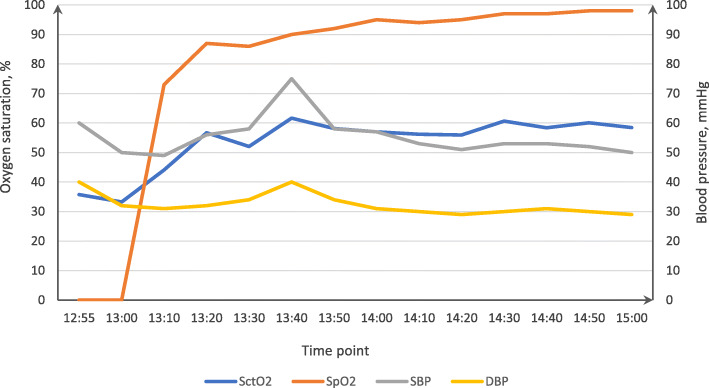
Readings of arterial blood pressure, SpO_2_ and SctO_2_ after cardiopulmonary bypass

Given the low blood pressure, low arterial blood oxygen saturation, and the low SctO_2_, poor cardiac output was considered as the likely cause. Therefore, the rate of epinephrine infusion was increased to 0.12 μg/(kg.min) and milrinone was infused intravenously at a rate of 1 μg/(kg.min). After 20 min of inotropic treatment, the SctO_2_ increased gradually from 35.7 to 61%, and photoplethysmography measurements from the finger and ear were now detectable and SpO_2_ increased to 98%, but notably without a significant increase in blood pressure. Given the acceptable cerebral tissue oxygen saturation and SpO_2_, no further treatment was performed to increase blood pressure. When the infant transferred to the pediatric intensive care unit, the SctO_2_ was about 56%, with a SpO_2_ of 99% and arterial blood pressure 48/29 mmHg. Epinephrine was infused at a rate of 0.1 μg/(kg.min) and milrinone at 0.5 μg/(kg.min). Arterial blood pressure, SpO_2_ and SctO_2_ readings are shown in Fig. 1. An infusion of 170 mL crystalloid fluid was administered and 100 mL urine was output during the course of general anesthesia (7.2 h). Because of severe myocardial swelling, the thorax was only closed 3 days later. This infant was followed-up 3 months later and no neurological complications had occurred.

## Discussion and conclusions

In this report, we have described a case of cerebral oximetry measurements during CPB weaning and the medication administered without available SpO_2_ or TEE assisted hemodynamic measurements in an infant who developed a severe low blood pressure. Even though little increase of blood pressure occurred after treatment with a high dose of epinephrine and milrinone, an acceptable level of SctO_2_ was achieved. After delayed sternal closure, the infant was discharged from hospital without any discernible neurological complications. This case, which integrated the practical use of cerebral oximetry, raised several important issues worthy of discussion.

Cerebral tissue is highly vulnerable during CPB for its weak tolerance to hypoxia, even for a short period of time. Thus, cerebral injury is a common complication after cardiac surgery [3, 4]. As an essential target organ of the systemic circulation, the monitoring status of cerebral oxygen metabolism is of great significance for evaluating hemodynamic management. Over the past few decades, ﻿near-infrared spectroscopy based regional SctO_2_ monitoring has been widely used to estimate the balance between oxygen supply and consumption in cerebral tissue to a depth of about 15 mm [5]. A decrease in SctO_2_ is associated with postoperative cerebral injury, neurocognitive dysfunction, a prolonged hospital stay and increased cost [6].

The value of SctO2 is determined by two main factors, oxygen supply and oxygen consumption [7]. Changes in cardiac outflow, mean arterial pressure (MAP), intracranial pressure (ICP), venous return and oxygen saturation of arterial blood, are considered to be the main factors that affect cerebral oxygen supply. The degree of aerobic metabolism of cerebral tissue is also associated with oxygen consumption. As demonstrated in previous studies, maneuvers including optimizing blood pressure and cardiac output, maintaining an appropriate head position, end-tidal CO_2_ and hemoglobin levels, are commonly used to augment cerebral oxygen supply. In addition, intravenous anesthesia, hypothermia or anti-epileptic medication, are also options to decrease oxygen consumption of cerebral tissue [5, 6, 8].

In the present case, the depth of anesthesia and the head position was unremarkable. Severe hypotension and myocardial edema were the main manifestations after CPD, accompanied by ﻿systemic hypoxia and a low SctO_2_. Given the severe myocardial edema, the reduction in blood pressure and SpO_2_ was mainly interpreted as being caused by a decrease in cardiac output. Therefore, epinephrine was first administrated, but had little effect on blood pressure [9]. Due to the low body weight, no available TEE probe could be used to understand further the etiology and to decide whether to continue cardiotonic therapy or administer norepinephrine to increase peripheral resistance. With SctO_2_ monitoring, 0.5–1 μg/kg milrinone was administered tentatively to increase cardiac output. Even though the blood pressure remained low after treatment, the SctO_2_ value was significantly increased.

It is likely that low blood pressure is an important cause of poor cerebral perfusion, based on the formula: cerebral perfusion pressure = mean blood pressure - intracerebral pressure. Increasing blood pressure is listed as the first parameter to consider for cerebral desaturation by proposed algorithms [5]. However, recent studies have revealed that a higher blood pressure did not produce an increase in SctO_2_. Holmgaard et al.*,* reported that at a fixed cardiopulmonary bypass pump flow rate of 2.4 L/min/m^2^, targeting a high MAP (70–80 mmHg) by norepinephrine produced more frequent and pronounced cerebral desaturation than a low MAP (40–50 mmHg) during bypass [10]. Similar findings were also reported in previous studies [11, 12]. The effect of increasing MAP by raising the SctO_2_ level remains to be debated. Several studies have also shown the discrepancy in the effects of epinephrine and other vasoconstrictors such as phenylephrine on SctO_2_ [13, 14]. Even though epinephrine and milrinone elevated SctO_2_ in the present case, the controversy remains to be resolved.

However, because the baseline level of SctO_2_ was not measured before anesthesia or surgery, the normal range or target of therapy for this infant was unclear. Multiple studies have been conducted to determine the normal range of SctO_2_ [15, 16]. Kussman et al. reported in children with congenital heart disease that SctO_2_ ranged from 56 to 82% with a median of 73%, which was affected by the hemoglobin concentration [15]. Nonetheless, SctO_2_ changes referring to baseline rather than absolute values are preferred in clinical practice or research studies [17, 18]. Furthermore, the present case indicated that systemic oxygen saturation is correlated with SctO_2_, shown by the parallel increase in SctO_2_ and SpO_2_. But it should be noted that a large discrepancy between SctO_2_ and ﻿somatic tissue oxygen saturation was demonstrated in previous studies in adult surgical patients [19].

In conclusion, for an infant with difficult CPB weaning, especially one with a low cardiac output resulting in SpO_2_ monitoring failure, cerebral oximetry is applicable for evaluating and directing hemodynamic management. Appropriate treatment is based on meticulous analysis among the variables of blood volume, cardiac function, mean blood pressure, hypoxia, anemia, and so on*.* A further study will be necessary to evaluate the effects vasoconstrictor-induced increase in blood pressure on SctO_2_ and the underlying mechanisms.

## Supplementary information

**Additional file 1: Table S1.** Measurements of arterial blood pressure, SpO_2_ and SctO_2_, also shown in Fig. 1.

**Additional file 2: Table S2.** Results of blood gas analysis during surgery. 08:12 anesthesia induced; 09:40 surgery started; 10:28 CPB started; 10:43 aorta cross-clamped; 12:11 aorta clamp released; 12:50 CPB ended; 15:25 surgery ended. A, arterial blood; V, venous blood.

## Data Availability

All data generated or analyzed during this study are included in this published article and its additional information file.

## Abbreviations

ASD: Atrial septal defect
CPB: Cardiopulmonary bypass
TEE: Transesophageal echocardiography
ICP: Intracranial pressure
MAP: Mean arterial pressure
NIRS: Near-infrared spectroscopy
PaCO_2_: Carbon dioxide partial pressure
PO_2_: Oxygen partial pressure
SctO_2_: Cerebral tissue oxygen saturation
SpO_2_: Pulse oxygen saturation
TTE: Transthoracic echocardiography
VSD: Ventricular septal defect
