# Supplementary material for: Severe low cerebral oximetry in difficult cardiopulmonary bypass weaning of low body-weight infant: a case report and literature review
Source: BMC Anesthesiol. 2020 Jun 27;20:159. doi: 10.1186/s12871-020-01071-1 (PMC7320539; doi:10.1186/s12871-020-01071-1)
Supplement: Supplementary file 1 — Additional file 1: Table S1. Measurements of arterial blood pressure, SpO2 and SctO2, also shown in Fig. 1. [file 12871_2020_1071_MOESM1_ESM.docx]

**Supplementary file**

**Table 1.** Measurements of arterial blood pressure, SpO_2_ and SctO_2_, also shown in Figure 1

|  | SctO_2_ (%) | SpO_2_ (%) | SBP (mmHg) | DBP (mmHg) |
| --- | --- | --- | --- | --- |
| 12:55 | 35.74 | * | 60 | 40 |
| 13:00 | 33.24 | * | 50 | 32 |
| 13:10 | 44.06 | 73 | 49 | 31 |
| 13:20 | 56.67 | 87 | 56 | 32 |
| 13:30 | 52.07 | 86 | 58 | 34 |
| 13:40 | 61.67 | 90 | 75 | 40 |
| 13:50 | 58.12 | 92 | 58 | 34 |
| 14:00 | 57.01 | 95 | 57 | 31 |
| 14:10 | 56.17 | 94 | 53 | 30 |
| 14:20 | 55.92 | 95 | 51 | 29 |
| 14:30 | 60.67 | 97 | 53 | 30 |
| 14:40 | 58.41 | 97 | 53 | 31 |
| 14:50 | 60.10 | 98 | 52 | 30 |
| 15:00 | 58.44 | 98 | 50 | 29 |
