# Supplementary material for: Severe low cerebral oximetry in difficult cardiopulmonary bypass weaning of low body-weight infant: a case report and literature review
Source: BMC Anesthesiol. 2020 Jun 27;20:159. doi: 10.1186/s12871-020-01071-1 (PMC7320539; doi:10.1186/s12871-020-01071-1)
Supplement: Supplementary file 2 — Additional file 2: Table S2. Results of blood gas analysis during surgery. 08:12 anesthesia induced; 09:40 surgery started; 10:28 CPB started; 10:43 aorta cross-clamped; 12:11 aorta clamp released; 12:50 CPB ended; 15:25 surgery ended. A, arterial blood; V, venous blood. [file 12871_2020_1071_MOESM2_ESM.docx]

**Supplementary file**

**Table 2.** Results of blood gas analysis during surgery.

| Time points | Sample | pH | PCO_2_  (mmHg) | PO_2_  (mmHg) | Hct  (%) | Na^+^  (mmol/L) | K^+^  (mmol/L) | Ca^2+^  (mmol/L) | Glu  (mmol/L) | Lac  (mmol/L) | Hb (g/L) | SO_2_  (%) | cHCO_3_^-1^  (mmol/L) | BE  (mmol/L) | Cl  (mmol/L) |
| --- | --- | --- | --- | --- | --- | --- | --- | --- | --- | --- | --- | --- | --- | --- | --- |
| 09:35 | A | 7.34 | 39.5 | 218.8 | 23.4 | 138.7 | 2.55 | 1.11 | 3.6 | 1.0 | 82.4 | 100 | 20.6 | -4.8 | 114.9 |
| 10:32 | A | 7.16 | 39.1 | 188.5 | 22.3 | 142.2 | 3.55 | 0.63 | 6.0 | 2.1 | 80.7 | 100 | 13.5 | -14.1 | 118.6 |
| 10:58 | A | 7.26 | 38.4 | 66.5 | 18.0 | 143.1 | 4.08 | 0.75 | 5.9 | 1.2 | 71.8 | 96.2 | 17.0 | -9.19 | 121.3 |
| 11:15 | A | 7.36 | 25.6 | 83.4 | 17.8 | 142.7 | 3.32 | 0.78 | 4.7 | 1.3 | 71.1 | 98.9 | 14.2 | -10.09 | 120.2 |
| 11:30 | V | 7.38 | 28.5 | 34.0 | 17.1 | 143.0 | 4.67 | 0.76 | 5.4 | 1.6 | 72.5 | 79.7 | 16.5 | -7.77 | 120.3 |
| 12:25 | V | 7.45 | 20.0 | 39.2 | 23.3 | 144.9 | 4.45 | 0.73 | 4.8 | 2.3 | 99.9 | 89.2 | 13.6 | -8.71 | 120.6 |
| 12:56 | A | 7.45 | 30.4 | 366.9 | 23.2 | 151.7 | 3.36 | 1.08 | 8.9 | 2.2 | 104.5 | 100 | 20.6 | -2.63 | 120.8 |
| 13:05 | A | 7.29 | 43.2 | 35.4 | 22.8 | 151.4 | 3.47 | 1.12 | 8.8 | 2.8 | 100.5 | 72.5 | 20.3 | -5.93 | 121.8 |
| 13:37 | V | 7.25 | 42.1 | 43.6 | 38.9 | 152.0 | 3.99 | 1.15 | 10.1 | 4.3 | 141.5 | 78.9 | 17.6 | -9.35 | 115.9 |

08:12 anesthesia induced; 09:40 surgery started; 10:28 CPB started; 10:43 aorta cross-clamped; 12:11 aorta clamp released; 12:50 CPB ended; 15:25 surgery ended. A, arterial blood; V, venous blood.
